# Long-term risk of reoperation after modular hemiarthroplasty: Any differences between uni- or bipolar design?

**DOI:** 10.1186/s12891-023-07035-z

**Published:** 2023-11-24

**Authors:** Dennis Lind, Jonatan Nåtman, Maziar Mohaddes, Cecilia Rogmark

**Affiliations:** 1 Department of Orthopedics, Skane University Hospital, Lund University, Lund, Sweden; 2 Swedish Arthroplasty Register, Centre of Registers Västra Götaland, Gothenburg, Sweden; 3 Department of Orthopedics, Institute of Clinical Sciences, Sahlgrenska Academy, University of Gothenburg, Gothenburg, Sweden

**Keywords:** Femoral neck fractures, Hemiarthroplasty, Bipolar, Unipolar, Dislocation, Periprosthetic Infection

## Abstract

**Background:**

It is unclear whether unipolar (UHA) or bipolar (BHA) hemiarthroplasty should be the preferred treatment of femoral neck fracture (FNF).

**Aim:**

We investigated the reoperation rate at 13 years post-fracture after BHA and UHA as treatment of FNF, including a subgroup analysis of individuals who survived 5 years or more, and described the reasons for reoperation after BHA and UHA respectively.

**Methods:**

In an observational cohort study on prospectively collected national register data, 16,216 BHA and 22,186 UHA were available for matching. A propensity score for treatment with bipolar HA was estimated using logistic regression. Matching was done using the 1:1 nearest neighbor matching without replacement. Of the 16,216 BHA patients, 12,280 were matched to a UHA control. A subgroup analysis based on the matched sample excluded individuals who died within 5 years and comprised 3,637 individuals with BHA and 3,537 with UHA. Kaplan-Meier survival analysis was used.

**Results:**

In the Kaplan-Meier analysis, 92% of the BHA group was free from reoperation at 13 years (95% CI 0.91–0.93), compared to 92% in the UHA group (CI 0.89–0.94). BHA was associated with more reoperations until 3 years. Reoperation due to infection was most common after BHA, n = 212 (1.7%) compared to n = 141 (1.1%) after UHA. Dislocation led to reoperation in 192 of the BHA cases (1.6%) and in 157 of the UHA cases (1.3%). Acetabular erosion/pain occurred in 0.1% and 0.4%. Amongst those surviving ≥ 5 years, 93% of the BHA group was free from reoperation (CI 0.92–0.94) at 13 years, 92% after UHA (CI 0.90–0.94). BHA had more reoperations during the 1st year only. The causes for reoperations showed similar rates except for acetabular erosion/pain. Here the BHA group had 2 cases (0.1%), the UHA had 39 (1.1%).

**Conclusion:**

With a modular hemiarthroplasty relatively few patients need a reoperation. During the first years, there is a higher reoperation rate after BHA compared to UHA. Thereafter, no differences are seen. In patients who survive ≥ 5 years after the fracture there are more reoperations due to acetabular erosion after UHA, but crude numbers are extremely low, and the total reoperation rate is not affected.

## Introduction

In the case of a displaced femoral neck fracture in an elderly individual, hip hemiarthroplasty (HA) is the most common treatment option [1]. In unipolar hemiarthroplasty (UHA) the articulation occurs between the prosthesis head and the acetabular cartilage, which over time may lead to acetabular erosion. The bipolar hemiarthroplasty (BHA) includes a second articulation, where an inner metallic head articulates within a polyethylene and metallic shell, which in turn articulates to the acetabular cartilage. By transferring much of the articulation to the inner bearing, the risk of developing acetabular erosion was thought to be reduced.

However, this has been difficult to show in clinical studies, and clinical equipoise still exist as whether the articulation in a hemiarthroplasty should be unipolar or bipolar. Metanalyses have shown similar results for BHA and UHA, with the exception of slightly less acetabular erosion with BHA [2, 3], A higher re-operation risk has been found for BHA compared to UHA in Sweden [4]. In contrast, the Australian National Joint Replacement Registry found BHA implants to have a lesser risk of revision compared to UHA [5]. Studies may be influenced by patient selection. When active patients are treated with UHA, erosion may be frequent [6, 7]. Finally, BHA may be associated with a higher risk of revision due to dislocation and infection [4, 8].

The proposed benefit of the dual articulation of the BHA to decrease acetabular wear has to be weighed against potential disadvantages [4]. Is there a difference in long-term implant survival between the UHA and BHA? Are UHA and BHA patients re-operated for the same reasons? As those patients who survive several years after their fracture are those that could potentially develop acetabular erosion, would they benefit from having a BHA?

### Aims

Our primary aim was to investigate any difference in the reoperation rate between BHA and UHA after treatment of femoral neck fractures, including a subgroup analysis of healthier individuals who had a survival of 5 years or more.

Our secondary aim was to describe the reasons for reoperation after BHA and UHA respectively.

## Patients and methods

This is an observational cohort study on prospectively collected data. 57,800 primary operations due to hip fracture were identified between 2005 and 2015 in the Swedish Arthroplasty Register (SAR). SAR has registered HA since 2005 and has for this procedure a 97% completeness [9]. In Sweden the orthopedic department most often chose on an administrative level to use BHA or UHA. Rarely is this left to the surgeon’s discretion. Thus, the choice of implant is to a lesser extent influenced by individual surgeon’s preference or patients’ comorbidities.

**Fig. 1:**
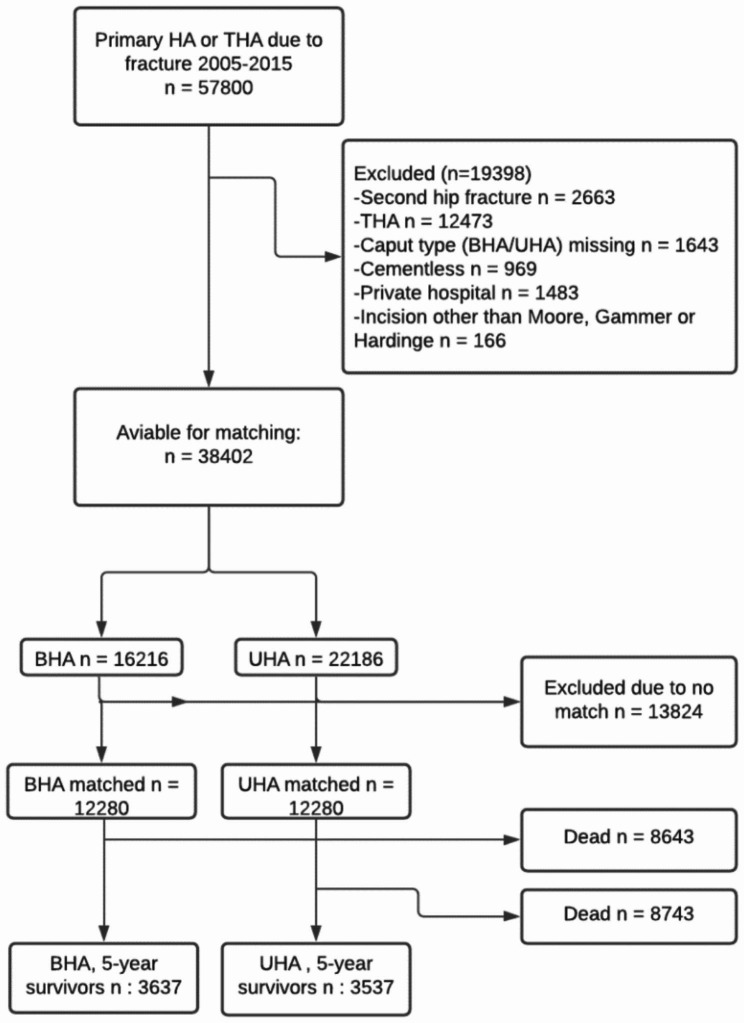
Flowchart describing eligible, excluded and included patients HA = hemiarthroplasty, THA = total hip arthroplasty, BHA = bipolar hemiarthroplasty, UHA = unipolar hemiarthroplasty

2,664 patients were treated with bilateral arthroplasties due to subsequent, bilateral fractures within the study period. If the patient had a bilateral surgery during the study period only the first surgery was included. Exclusion criteria were missing information on type of head (BHA/UHA), type of approach, cement less design and surgery in private hospitals. Surgeries reported in 2015 were excluded to allow a minimum 1-year follow-up. This led to 16,216 BHA and 22,186 UHA that were available for matching (Fig. 1).

Our primary outcome reoperation is defined as any secondary open surgery in the injured hip reported to SAR.

### Statistics

#### Matching

A propensity score for treatment with bipolar HA was estimated using logistic regression. The following baseline variables were included in the model: Year of surgery, age, approach, sex, and hospital type.

Matching was done using the 1:1 nearest neighbor matching without replacement. A caliper of 0.1 standard deviations of the logit of the propensity score was used. Analyses in the matched cohort provides estimates of the average treatment effect of bipolar HA compared to unipolar HA, on the patients who did receive the treatment (ATT). The ability of the PS matching to balance the baseline covariates was assessed using absolute standardized mean differences (SMD). A SMD < 10% was considered non-significant.

Of the 16,216 patients treated with bipolar HA, 12,280 patients were matched to a unipolar HA control within the specified caliper distance. 3,936 patients (24%) were excluded.

#### Subgroup analysis

To depict the outcome in less frail individuals with a theoretical higher activity level at the time of fracture, a subgroup analysis was conducted including only those patients who had at least 5 years of follow-up time, i.e. individuals who died within 5 years from the index surgery are excluded. The analysis was based on the matched sample and comprised of 3,637 individuals with BHA and 3,537 with UHA (30% and 2% of the total matched sample, respectively).

#### Survival analysis

Kaplan-Meier survival analysis was used for the different subgroup analysis, and any reoperation was set as an event. R version 3.6.1 was used for all statistical analyses.

### Ethics, funding, and potential conflicts of interest

The study was approved by the Regional Ethical Review Board in Gothenburg, Sweden (ref. 882 − 17) and the the Swedish National Ethical Review Board (ref. 2019–05024). Patients are informed about registration in the SAR at the time of their arthroplasty procedure and have the possibility to decline participation. As the patient information also mentions that register data can be used in research, no further informed consent is necessary. The study adhered to the Helsinki Declaration and is reported based on the STROBE guidelines.

This work was supported by grants from the Southern Health Care Region, Sweden. It was also funded by an ALF grant (Swedish Research Council funding for clinical research in medicine). The funding bodies had no involvement in the conduction of the study or the preparation of the article. No competing interests were declared.

## Results

### Demographics

Before matching, the mean age and the ASA class of the BHA group was lower, whilst surgery during the early study period, posterior approach and women were overrepresented (Table 1). After matching, these differences were less pronounced. In particular, there were no longer differences regarding sex, surgical approach and year of surgery (Table 1).

**Table 1:** Baseline characteristics for the overall cohort and the matched cohort

		Overall cohort					Matched cohort				
		Bipolar	Unipolar	*p*	SMD	Missing (%)	Bipolar	Unipolar	*p*	SMD	Missing
Number of patients		16,216	22,186				12,280	12,280			
Mean age (SD)		83.81 (6.75)	84.47 (6.81)	< 0.001	0.098	0.0	84.53 (6.80)	83.95 (6.95)	< 0.001	0.085	0.0
Mean BMI (SD)		23.93 (4.36)	23.68 (3.97)	0.001	0.060	62.8	23.91 (4.37)	23.64 (4.03)	0.004	0.063	66.0
Age group - years (%)	< 75	1473 ( 9.1)	1770 ( 8.0)	< 0.001	0.096	0.0	929 ( 7.6)	1109 ( 9.0)	< 0.001	0.062	0.0
	> 85	6970 (43.0)	10,579 (47.7)				5731 (46.7)	5453 (44.4)			
	75–85	7773 (47.9)	9837 (44.3)				5620 (45.8)	5718 (46.6)			
Sex (%)	Men	4709 (29.0)	6970 (31.4)	< 0.001	0.052	0.0	3497 (28.5)	3627 (29.5)	0.070	0.023	0.0
	Women	11,507 (71.0)	15,216 (68.6)				8783 (71.5)	8653 (70.5)			
Year of surgery 2 (%)	2005–2006	3537 (21.8)	2383 (10.7)	< 0.001	0.584	0.0	2199 (17.9)	2101 (17.1)	0.514	0.023	0.0
	2007–2008	4294 (26.5)	2746 (12.4)				2447 (19.9)	2508 (20.4)			
	2009–2010	3025 (18.7)	4376 (19.7)				2397 (19.5)	2434 (19.8)			
	2011–2012	2438 (15.0)	4908 (22.1)				2322 (18.9)	2322 (18.9)			
	2013–2015	2922 (18.0)	7773 (35.0)				2915 (23.7)	2915 (23.7)			
Approach (%)	Posterior	8715 (53.7)	6494 (29.3)	< 0.001	0.513	0.0	5384 (43.8)	5212 (42.4)	0.028	0.028	0.0
	Lateral	7501 (46.3)	15,692 (70.7)				6896 (56.2)	7068 (57.6)			
Hospital type (%)	University or regional	4677 (28.8)	6037 (27.2)	< 0.001	0.121	0.0	3908 (31.8)	4183 (34.1)	< 0.001	0.066	0.0
	County	9393 (57.9)	12,250 (55.2)				6915 (56.3)	6856 (55.8)			
	Rural	2146 (13.2)	3899 (17.6)				1457 (11.9)	1241 (10.1)			
ASA class (%)	1	301 ( 3.3)	340 ( 1.9)	< 0.001	0.129	30.6	242 ( 3.0)	186 ( 2.2)	< 0.001	0.101	33.4
	2	3524 (38.3)	6189 (35.5)				3001 (37.6)	3044 (36.3)			
	3	4931 (53.6)	9719 (55.7)				4350 (54.6)	4589 (54.7)			
	4	451 ( 4.9)	1205 ( 6.9)				380 ( 4.8)	571 ( 6.8)			

Bipolar = bipolar hemiarthroplasty, Unipolar = unipolar hemiarthroplasty SMD = Standardized mean difference, SD = Standard deviation, BMI = body mass index

### Matched cohort

**Fig. 2:**
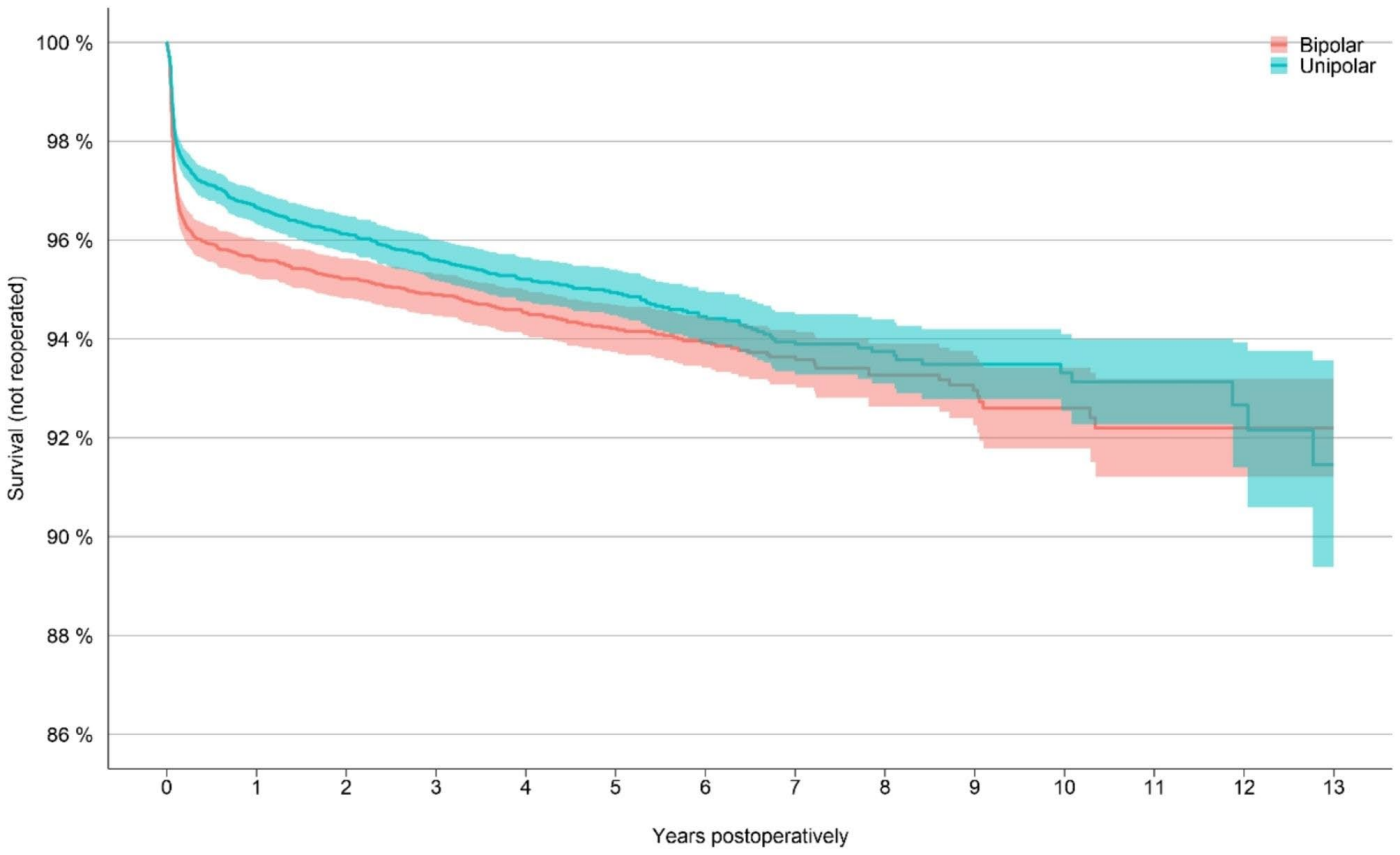
Kaplan-Meier analysis for the matched cohort, survival referring to patients not being reoperated Bipolar = bipolar hemiarthroplasty, unipolar = unipolar hemiarthoplasty

In the matched cohort with 12,280 in each group, 603 (4.9%) were re-operated after BHA and 476 (3.9%) after UHA the end of the follow-up period (Table 2). BHA was associated with more early reoperations, but already after 3 years we observe no significant difference in the rate of reoperation (Fig. 2).

**Table 2:** -The different reasons for the first reoperation for the matched cohort and for the subgroup of those surviving more than 5 years after the fracture

	Matched cohort				5-year survivors			
	Bipolar n = 12,280		Unipolar n = 12,280		Bipolar n = 3,637		Unipolar n = 3,537	
Reason	n	%	n	%	n	%	n	%
No reoperation	11,677	95.1	11,804	96.1	3,446	94.7	3,371	95.3
Infection	212	1.7	141	1.1	57	1.6	37	1.0
Dislocation/instability	192	1.6	157	1.3	57	1.6	39	1.1
Femoral fracture	155	1.3	107	0.9	57	1.6	42	1.2
Other	18	0.1	13	0.1	7	0.2	1	0.0
Aseptic loosening	17	0.1	9	0.1	10	0.3	8	0.2
Acetabular erosion/pain	7	0.1	48	0.4	2	0.1	39	1.1
Other technical failure	2	0.0	1	0.0	1	0.0	0	0

In the Kaplan-Meier analysis, 92% of the BHA group was free from reoperation at 13 years (95% CI 0.91–0.93), compared to 92% in the UHA group (95% CI 0.89–0.94). Numbers at risk were 102 and 111, respectively.

### Subgroup analysis of individuals with a minimum of 5 years survival

**Fig. 3:**
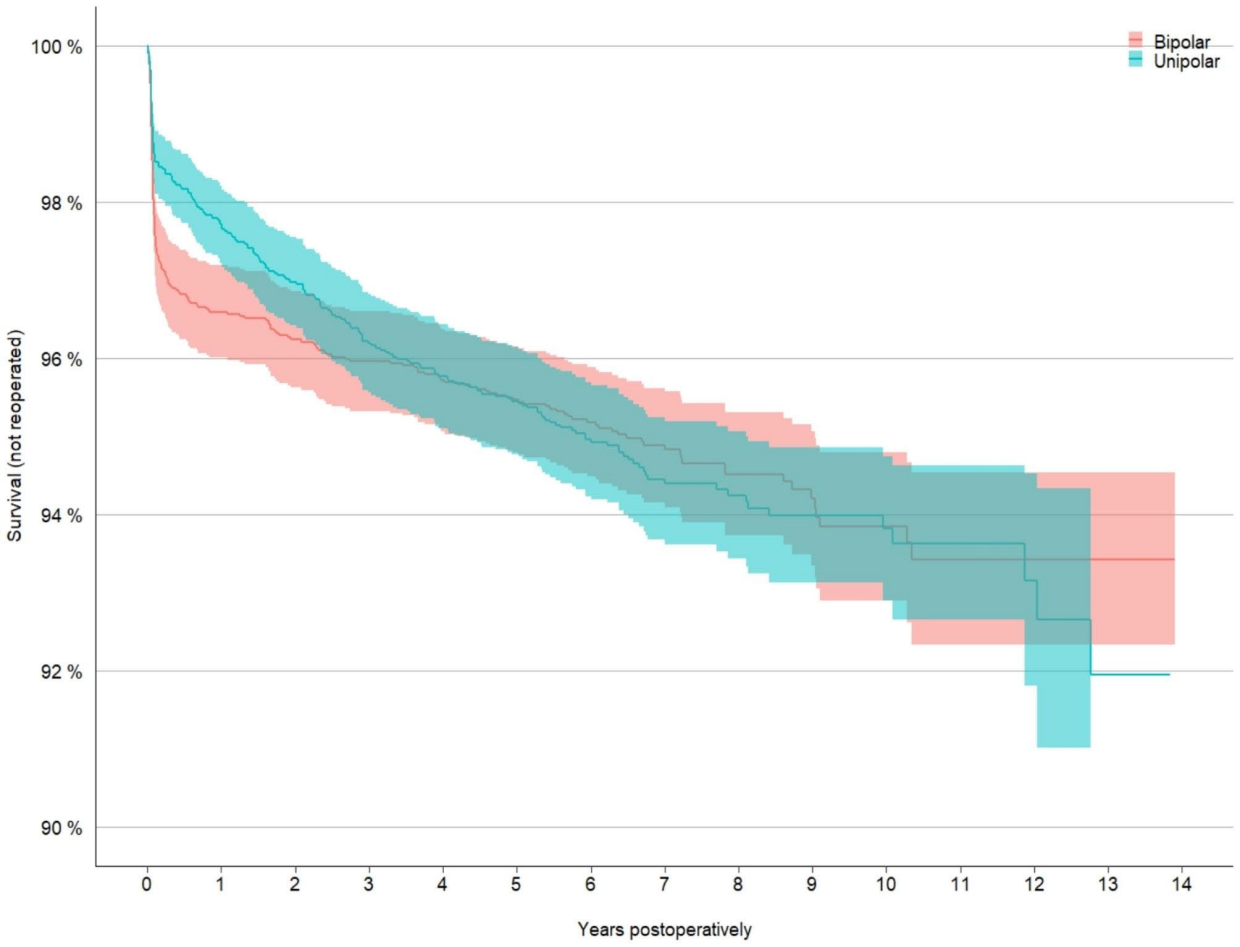
Kaplan -Meier subgroup analysis of the 5-year survivors, survival referring to patients not being reoperate Bipolar = bipolar hemiarthroplasty, unipolar = unipolar hemiarthoplasty

Groups were similar in most aspects (Table 3). The reoperation rate for individuals who survived 5 years after their hip fracture was 192/3637 (5.3%) in the BHA and 166/3537 (4.7%) in the UHA group. Also in this subgroup analysis an early difference between the groups was observed, but after the first year there was no significant difference in the rate of reoperation (Fig. 3).

**Table 3:** Baseline characteristics for the 5-year survivor group

		Bipolar	Unipolar	*P* Value	SMD	Missing
n		3637	3537			
Mean age (SD)		81.92 (6.52)	81.89 (6.73)	0.827	0.005	0.0
Mean BMI (SD)		24.57 (4.46)	24.49 (4.03)	0.642	0.019	66.3
Age group - years (%)	< 75	440 (12.1)	451 (12.8)	0.231	0.040	0.0
	> 85	1072 (29.5)	1090 (30.8)			
	75–85	2125 (58.4)	1996 (56.4)			
Sex (%)	Men	731 (20.1)	707 (20.0)	0.931	0.003	0.0
	Women	2906 (79.9)	2830 (80.0)			
Year of surgery (%)	2005–2006	778 (21.4)	777 (22.0)	0.804	0.030	0.0
	2007–2008	781 (21.5)	754 (21.3)			
	2009–2010	771 (21.2)	771 (21.8)			
	2011–2012	731 (20.1)	708 (20.0)			
	2013–2015	576 (15.8)	527 (14.9)			
Approach (%)	Posterior	1690 (46.5)	1660 (46.9)	0.710	0.009	0.0
	Lateral	1947 (53.5)	1877 (53.1)			
Hospital type (%)	University or regional	1063 (29.2)	1204 (34.0)	< 0.001	0.108	0.0
	County	2060 (56.6)	1902 (53.8)			
	Rural	514 (14.1)	431 (12.2)			
ASA class (%)	1	122 (5.6)	88 (4.0)	0.036	0.088	38.5
	2	1112 (50.8)	1107 (49.8)			
	3	910 (41.6)	976 (43.9)			
	4	43 (2.0)	53 (2.4)			

Bipolar = bipolar hemiarthroplasty, Unipolar = unipolar hemiarthroplasty SMD = Standardized mean difference, SD = Standard deviation, BMI = body mass index

At 13 years, 93% of the BHA group was free from reoperation (95% CI 0.92–0.94), compared to 92% in the UHA group (95% CI 0.90–0.94).

### Reoperation for specific reasons

In the entire matched cohort, BHA and UHA had the same major causes for reoperation. Infection was most common after BHA, n = 212 (1.7%) compared to n = 141 (1.1%) after UHA. Dislocation led to reoperation in 192 of the BHA cases (1.6%) and in 157 of the UHA cases (1.3%). Periprosthetic fracture occurred in 1.3% and 0.9%, acetabular erosion/pain in 0.1% and 0.4%. (Table 2).

A similar pattern was seen in subgroup analysis with long-term survival, except for reoperations due to acetabular erosion or pain. Here the BHA group had 2 cases (0.1%) the UHA had 39 (1.1%) (Table 2).

### Mortality

**Fig. 4:**
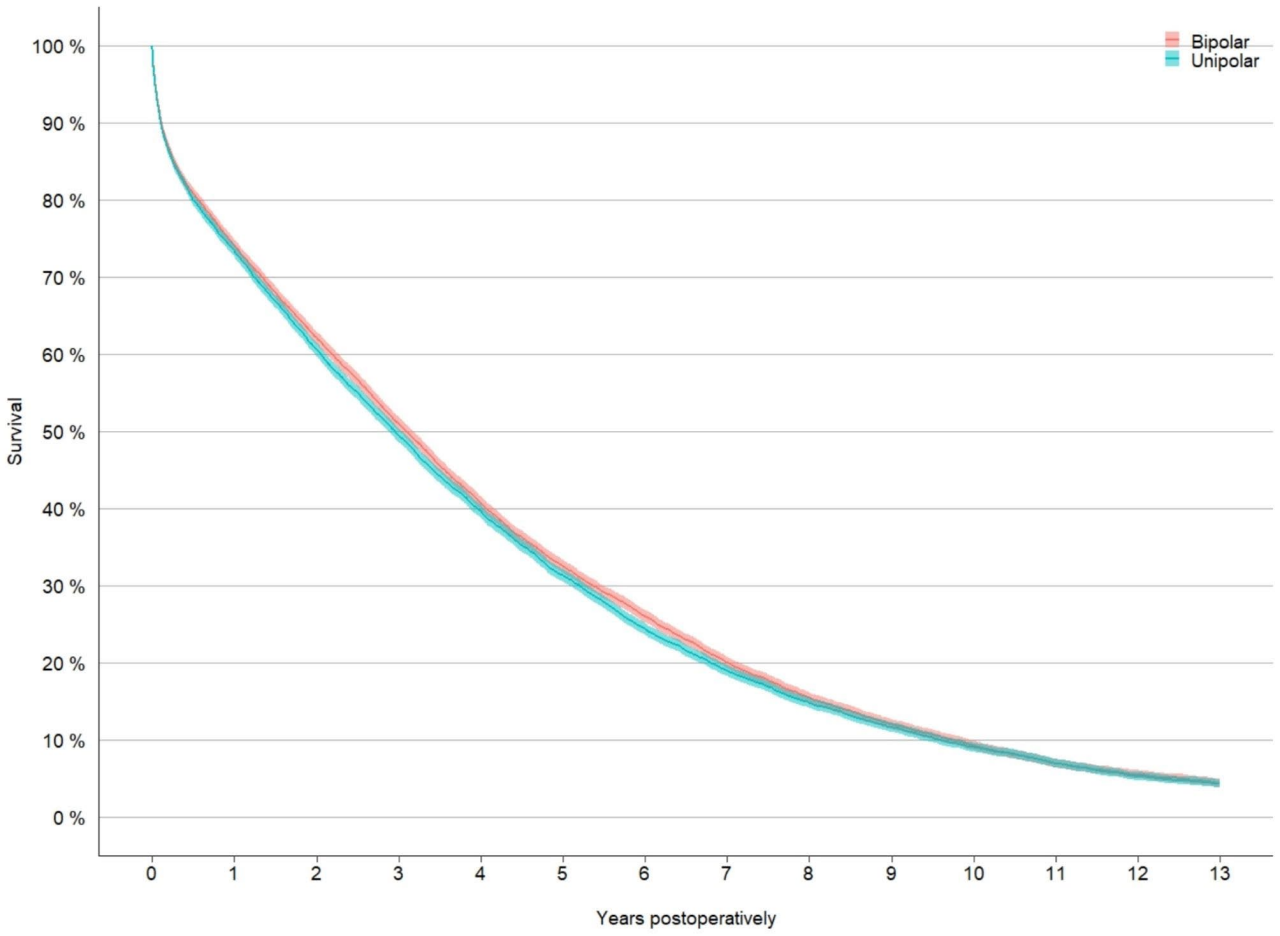
Kaplan -Meier analysis of the mortality in the matched cohort Bipolar = bipolar hemiarthroplasty, unipolar = unipolar hemiarthoplasty

A Kaplan-Meier survival analysis showed 1-, 5- and 10-years mortality rates of 26%, 68% and 91% after BHA. The results in the UHA group were 27%, 69% and 91% (Fig. 4).

**Table 4:** The rate of survivors after 1, 5 and 10 years

	Bipolar hemiarthroplasty		Unipolar hemiarthroplasty	
Year after fracture	Rate of survivors	CI	Rate of survivors	CI
1	0.74	0.73–0.75	0.73	0.73–0.74
5	0.32	0.32–0.33	0.31	0.31–0.32
10	0.09	0.09–0.10	0.09	0.08–0.10

## Discussion

In a matched cohort with a long-term follow-up, we found more early reoperations after bipolar hemiarthroplasty compared to unipolar. This difference was not measurable past 2 years and up to 13 years post-fracture, UHA and BHA had the same rate of reoperation.

To identify a subgroup of patients that would theoretically benefit from receiving a BHA, we selected the patients who survived 5 years or more after their fracture and performed a subgroup analysis. Neither in this assumingly healthier and more active group were there benefits for BHA in terms of fewer reoperations.

Regardless of implant type, patients in general were reoperated for three major reasons – infection, dislocation, and periprosthetic fracture. In those with a long survival the pattern was slightly different; for the patients operated with UHA reoperation due to acetabular erosion was just as common as these three aforementioned causes. Dislocation is known to occur within the first few months, regardless of whether BHA or UHA is used [10–13].

We confirm the earlier findings from Sweden that the UHA is associated with a lower early reoperation / revision rate compared to the BHA [4]. Our long term results are supported by a recent meta-analyses that found a higher rate of acetabular erosion after UHA but no significant differences in revision rate, dislocation or Harris Hip Score (HHS) [2, 3]. The included studies were however heterogenous and included outdated monoblock implants.

Our results are well aligned with data from a recent register study from Australia, showing few differences between UHA and BHA [14]: UHA had lower incidence of periprosthetic fractures and higher rates of revision due to erosion compared to BHA. The rate of revision for infection and dislocation did not differ between groups. They found similar revision rates overall for the both the BHA and UHA, around 3% over a 5-year period. As they reported on revisions only, our higher rate of reoperation may be explained by us using any open secondary surgery as outcome. Comparisons are hampered by different patient selection. The average patient registered in Australia is younger than his/her Swedish counterpart. Assumingly, a younger and more active person has a higher risk of developing acetabular erosion [15], and will subsequently more often be a candidate for secondary surgery, thanks to better health and longer remaining lifespan, resulting in more reoperations due to erosion [14]. In general, the implant types display a similar distribution of reoperation causes. One should bear in mind that the frailty of these patients may interfere with the decision to perform secondary surgery when a complication occurs. Even though all easily exchangeable parts should be replaced during a DAIR procedure when deep infection, a surgeon who stand in front of a very frail patient might choose to leave an all-metal unipolar head and treat a suspected infection non-operatively but be more prone to surgically exchange the polyethylene parts in a bipolar head. Even if a number of individuals with longer survival and UHA treatment were actually re-operated due to acetabular erosion, it did not lead to a detectable difference in the general reoperation rate compared to BHA. Comparing the incidence of acute conditions such as infection, dislocation, and fracture, with the gradually developing and initially clinical silent acetabulum erosion is problematic. We can assume that old and frail patients with deep infection, recurrent dislocations or periprosthetic fracture firstly will get attention from an orthopedic surgeon, and secondly more often have secondary surgery, compared to those with acetabulum erosion. The latter condition does not present as an acute threat to the patient’s health and life, and sedentary behavior will reduce the patient’s discomfort. Thus, symptomatic acetabular erosion is probably more common than studies focusing only on reoperation only can detect. In a randomized trial, Inngul et al. found a difference in health-related quality of life in favor of the bipolar head after 4 years, but no clinical difference in HHS between the two implants, nor any difference in reoperation rate [16].

### Strengths and limitations

As this study is register-based, conclusions regarding causality cannot be drawn. We are limited by the variables in the register, meaning that information on certain possible confounders, such as comorbidities, frailty and activity level is lacking. Neither do we have data on non-surgically treated complications, radiological outcome, or patient-reported outcome. We acknowledge that the absence of a reoperation does not equal a successful post-fracture recovery. Despite the good completeness of primary procedures and revisions to the SAR, we are aware of some underreporting of reoperations to the register. Still, we expect this possible underreporting to apply to both implant types equally. The somewhat higher risk of periprosthetic fractures in association with BHA is more of a stem problem, where certain common combinations of anatomically shaped stems and UHA might have biased our results. Our study is one of the largest to analyze differences in the long-term reoperation rate between UHA and BHA. The completeness and national coverage of the SAR are high. In Sweden the use of either UHA or BHA are decided on a departmental level. This, and the matched design of the study, renders comparable study groups, which also is underpinned by the similar mortality rates. We consider the external validity to be good, as all patients, regardless of cognitive function, and all surgeons and hospitals are included. Reoperation of any kind was chosen as the primary outcome, and not revision surgery, as secondary surgery in the frail often is limited to minor procedures.

## Conclusion

With a modern modular hemiarthroplasty after femoral neck fracture relatively few patients need a reoperation. During the first years, there is a higher reoperation rate after BHA compared to UHA, but thereafter, no differences are seen. In patients who survive more than 5 years after the fracture there are more reoperations due to acetabular erosion after UHA, but crude numbers are extremely low, and the total reoperation rate is not affected.

## Data Availability

The data that support the findings of this study are available from The Swedish Arthroplasty Register but restrictions apply to the availability of these data, which were used under license for the current study, and so are not publicly available. Case level dataset generated and analysed during the current study are not publicly available due regulation by Swedish authorities. Data are however available from the authors upon reasonable request and with permission of The Swedish Arthroplasty Register.
